# Cost-effectiveness of surgical and conservative interventions for orthopaedic injuries in Malawi: a modelling study

**DOI:** 10.1136/bmjgh-2025-022014

**Published:** 2026-08-14

**Authors:** Pakwanja Desiree Twea, Peter Hangoma, Ole Frithjof Norheim, Sven Young, Levison Chiwaula, Boston Munthali, David Watkins

**Affiliations:** 1 Bergen Centre for Ethics and Priority Setting in Health, University of Bergen, Bergen, Norway; 2 Department of Planning and Policy Coordination, Ministry of Health, Lilongwe, Malawi; 3 Chr Michelsen Institute, Bergen, Norway; 4 Department of Health Policy and Management, University of Zambia, Lusaka, Zambia; 5 Harvard T H Chan School of Public Health, Boston, Massachusetts, US; 6 Lilongwe Institute of Orthopaedics and Neurosurgery, Lilongwe, Malawi; 7 Department of Surgery, Haukeland Universitetssjukehus, Bergen, Norway; 8 Department of Economics, University of Malawi, Zomba, Malawi; 9 Division of General Internal Medicine, Department of Medicine, University of Washington, Seattle, Washington, USA; 10 Department of Global Health, University of Washington, Seattle, Washington, USA

**Keywords:** Orthopedic surgery, Health economics, Africa South of the Sahara, Treatment, Injury

## Abstract

**Background:**

Orthopaedic injuries are a major contributor to morbidity and mortality in low-income settings, driven by rising urbanisation and inadequate road safety measures. This study evaluates the cost-effectiveness of treatment for four common orthopaedic injuries in Malawi: femoral shaft fractures, patellar fractures, humeral shaft fractures and paediatric supracondylar humeral fractures (PSCHF).

**Methods:**

We developed a dynamic state-transition model to simulate fatal and non-fatal outcomes from a healthcare perspective from 2021 to 2031, informed by cost, epidemiological and population estimates. We estimated incremental cost-effectiveness ratios (ICER) for conservative and surgical management versus no treatment and each other, conducting one-way and probabilistic sensitivity analyses. We then modelled the population impact of scaling up the most cost-effective intervention under two scenarios.

**Findings:**

For femoral shaft fractures, surgical, conservative and no treatment resulted in disability-adjusted life years (DALYs) of 0.10, 0.21 and 0.23, with ICERs of approximately US$3000 and US$60 000 for surgical and conservative treatment. For patellar fractures, DALYs were 0.20, 0.22 and 0.32, with similar cost-effectiveness (ICERs of US$1400). For humeral shaft fractures, DALYs were 0.041, 0.056 and 0.11, with ICERs of US$3000 and US$1400. For PSCHF, DALYs were 0.04, 0.086 and 0.14, with ICERs of US$1600 and US$2600. Comparison of surgical and conservative strategies shows surgery was cost-saving for femoral shaft fractures, similar for patellar fractures, less efficient for humeral shaft fractures and more cost-effective for PSCHF. At Malawi’s willingness-to-pay threshold of US$65 per DALY averted, the probability of cost-effectiveness was low. Under the ambitious scale-up scenario, approximately 1800 DALYs (a 10% reduction relative to the baseline) were averted at an incremental cost of US$5 million.

**Conclusion:**

In Malawi, surgery is more cost-effective for femoral shaft and PSCHF fractures, whereas conservative care is more cost-effective for humeral shaft fractures. For patella fractures with an intact extensor mechanism, both treatments are similarly cost-effective. Targeted scale-up could yield substantial health gains, underscoring the importance of context-specific data for prioritising orthopaedic services in low-resource settings.

**WHAT IS ALREADY KNOWN ON THIS TOPIC**

- In settings with mature surgical systems, surgical intervention for fractures is often more cost-effective, less costly in the long run and associated with better functional outcomes than conservative management.
- Evidence on the cost-effectiveness of fracture treatment options in low-income countries is lacking, and no studies have compared outcomes across different fracture types in such settings.

**WHAT THIS STUDY ADDS**

- This is the first study from a low-resource setting to assess the cost-effectiveness of multiple fracture types within a single analysis.
- It provides national-level estimates of the cost-effectiveness of treatment choices for common orthopaedic fractures in Malawi.
- The study quantifies the potential health and economic gains from expanding treatment capacity, offering actionable evidence for priority setting.

**HOW THIS STUDY MIGHT AFFECT RESEARCH, PRACTICE OR POLICY**

- Demonstrates the importance of locally generated cost-effectiveness evidence to guide treatment decisions in resource-constrained settings.
- Shows how baseline treatment mix and capacity expansions can shift the relative risks and benefits of surgical versus conservative management.
- Provides policymakers with a framework to prioritise fracture types and interventions that maximise population health impact within limited budgets.

## Introduction

 In 2019, injuries accounted for approximately 7.6% of global deaths and 9.8% of disability-adjusted life years (DALYs) worldwide.^1^ Orthopaedic trauma, primarily resulting from transport-related and other unintentional injuries, constitutes a substantial portion of this burden, particularly in low- and middle-income countries.^1^ However, these countries are often the least equipped to respond due to limited surgical capacity, inadequate or non-existent pre-hospital emergency systems and greater inadequacies in the health infrastructure.^2 3^

These limitations have compounding effects on health systems in low-income countries, where the actual national burden and distribution of orthopaedic injuries and fractures are often unknown due to the lack of large-scale epidemiological studies, nationally representative trauma registries and reliable reporting mechanisms.^4 5^ Additionally, many patients are unable to access treatment due to supply and demand constraints, including long distances to healthcare facilities and financial barriers.^5^ The treatment options among those who do receive care are generally classified as either conservative management or surgical intervention. The choice between surgery and conservative treatment often depends on several factors, including the type and severity of the injury, the timing of healthcare-seeking and the healthcare system capacity.^6^ Health system constraints often make conservative management the default choice for many patients in LMICs, though the reality is that many individuals with injuries lack access to hospitals and other emergency services and therefore receive no treatment at all.

While conservative management is more commonly implemented in low-income settings, economic evaluations have generally found surgical treatment to be superior, offering higher success rates, fewer complications, faster recovery and in some cases lower overall costs.^7–9^ However, most existing studies focus on single fracture types, with limited attention to other injuries or to how the current distribution of treatment options affects population-level outcomes. Broader gaps in the literature have been documented, including limited evidence on the long-term consequences of treatment decisions and the economic burden of untreated injuries, particularly for upper extremity fractures.^10 11^ This study builds on the existing literature by estimating the medium-term health and financial consequences of untreated fractures and evaluating how the current distribution of treatment options shapes outcomes across four key fracture types: femoral shaft, humeral shaft, paediatric supracondylar humeral (PSCHF) and patellar fractures.

This study uses Malawi as a case study to characterise the cost-effectiveness of treatment strategies for alternative orthopaedic fractures managed at tertiary hospitals. We assess three approaches: no treatment, conservative management and surgical intervention. First, we estimate the cost-effectiveness of surgical and conservative treatment compared with no treatment for the four fracture types. Then, we evaluate the health and budget impact of incrementally expanding treatment capacity beyond current levels. Findings are intended to inform prioritisation decisions and provide evidence for similar low-income contexts.

## Methods

### Analytical overview

A dynamic population model (DPM) was developed to estimate the epidemiological, economic and cost-effectiveness impacts of treatments for the four types of orthopaedic fractures. The model compares three treatment strategies for fractures in the general population: no treatment (those who do not present to the hospital for treatment), informed by estimates from Ngoie *et al*^12^ and supported by evidence that a proportion of patients in similar settings do not seek care for fractures,^13^ conservative management and surgical intervention. The comparator treatments for conservative and surgical management were defined for each fracture type. For femoral shaft fractures, we compared skeletal traction with intramedullary nailing; for humerus and patellar fractures, casting with open reduction and internal fixation; and for PSCHF, casting with closed reduction and percutaneous pinning. The choice of comparator treatments reflects the most commonly used options reported in the literature and in observations from our previous costing study. Given the lack of clear norms regarding the distribution of specific surgical techniques in this setting, we selected representative treatments for each strategy. The comparison between conservative and surgical management reflects a key policy question for Malawi, where limited surgical capacity and resource constraints require prioritisation of cost-effective treatment strategies.

The model adopts a health system perspective and focuses on Malawi’s tertiary-level health system. This perspective was selected to reflect the scope of available cost and utilisation data and to align with the policy context in which resource allocation decisions are made. Fracture incidence in the model is adjusted to reflect the proportion of cases managed at the tertiary level, scaled down to 60% based on epidemiological trends observed by Schade *et al*.^14^ This treatment proportion is assumed to be constant across all fracture types. Costs were expressed in US dollars, and health effects were measured in DALYs, both discounted at an annual rate of 3% in line with World Health Organisation-CHOosing Interventions that are Cost Effective (WHO-CHOICE) guidelines for cost-effectiveness analysis.^15^ In this study, an intervention was considered cost-effective if its incremental cost-effectiveness ratio (ICER) fell below Malawi’s official willingness-to-pay (WTP) threshold of 65 USD per DALY averted.^16^ 2021 was selected as the base year to align with the available cost, effectiveness and epidemiological data.

### Model structure

**Figure 1:**
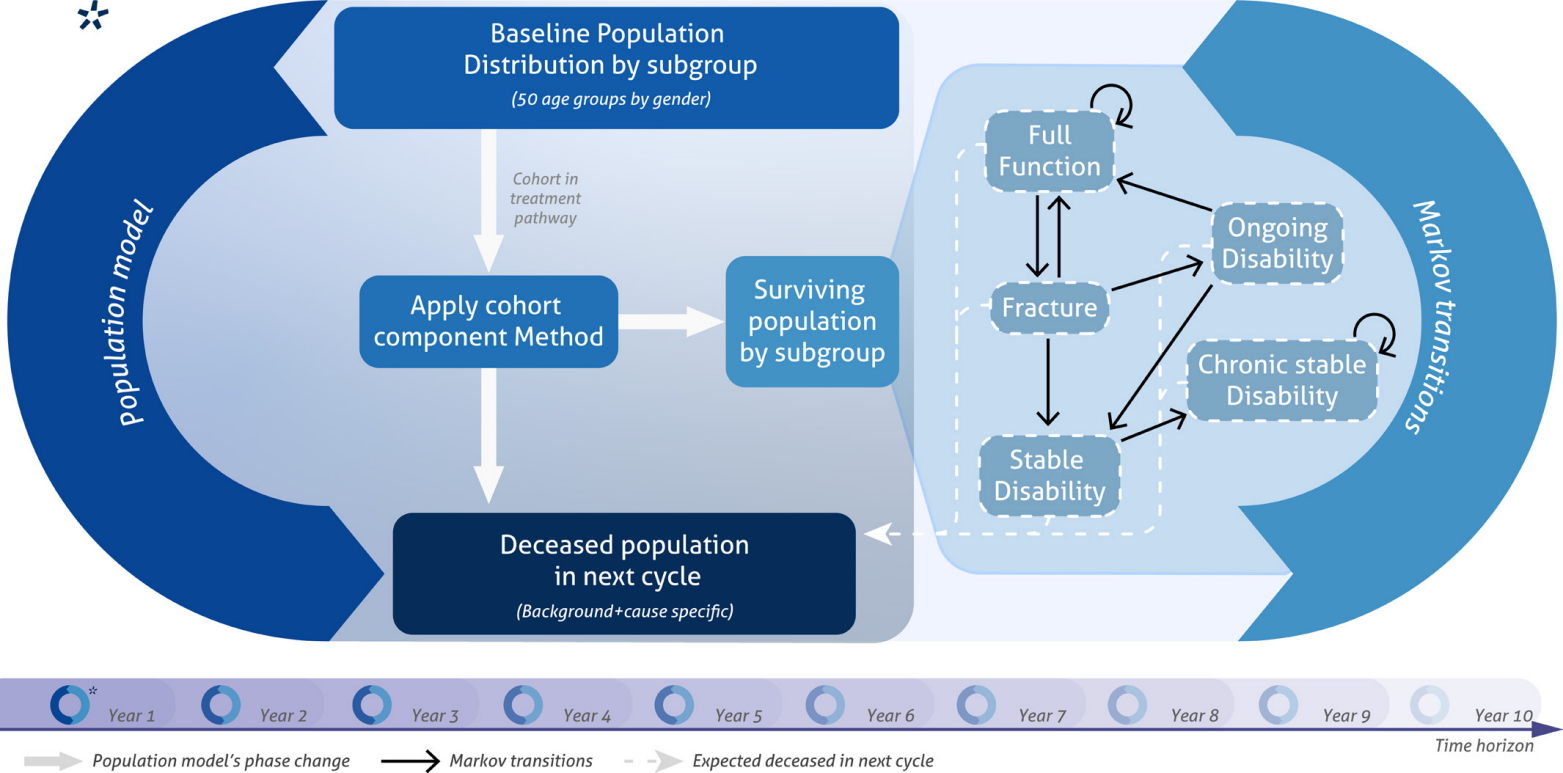
Conceptual schematic of the dynamic population model, illustrating the integration of population and epidemiological components of the model.

The DPM model has two submodels: the population part, which uses the cohort component method for forecasting and the epidemiological part, a Markov state transition model. The model uses 6-month time steps (cycle lengths) over a 10-year time horizon from 2021 to project medium-term outcomes. The 6-month cycle duration was selected to reflect the variability in fracture recovery periods and the time required to assess and definitively determine treatment outcomes. The 10-year horizon was selected to capture the medium-term health and economic consequences of treatment, including mortality, disability and recurrent costs. Figure 1 represents a schematic of the DPM. The model was developed and analysed using R software V.4.4.1^17^ and builds on code from Krijkamp *et al*^18^ and Green *et al*.^19^

The Markov (epidemiological) component of the model has seven possible health states: ‘Full function’, ‘Fracture’, ‘Ongoing Disability’, ‘Stable Disability’, ‘Chronic Stable Disability’, ‘Cause-Specific Death’ and ‘Background Death’. Orthopaedic clinicians were consulted in defining the relevant health states within the Markov model to ensure that patient pathways reflected realistic clinical practice in the Malawian context. Refer to box 1 for detailed definitions of health states.

Box 1 **Health state descriptions**

**State descriptions**

**Full** **function**

This is the initial state in which patients enter the model. It is also the state where patients who regain full functional capacity following a fracture return.

**Fracture**

This state represents individuals with a bone fracture that may require further medical treatment.

**Ongoing** **disability**

This is a tunnel state representing individuals who, after initial fracture treatment, continue to experience complications and remain eligible for further care. These individuals may require additional medical attention, physical therapy or revision surgery. Based on elicited probabilities, a proportion receive second-line interventions, such as repeat surgery or surgical conversion following initial conservative management, while others do not, due to clinical or health system constraints. Patients who receive second-line treatment either transition to full function if treatment is successful or to stable disability if it is unsuccessful. Those who do not receive further treatment also transition to stable disability.

**Stable disability**

This tunnel state represents individuals with permanent or long-term disabilities who have reached a stable condition and are no longer progressing. All individuals remain in this state for one cycle before transitioning to chronic stable disability.

**Chronic stable disability**

This state represents individuals previously in the stable disability state and is used to track chronic disability.

**Cause-specific death**

Individuals in this state have died from complications directly associated with their bone fractures.

**Background death**

This state represents individuals who have died from causes unrelated to their bone fracture.

The population component forecasts demographic change across 100 cohorts defined by sex and single-year ages (0–49), simulating individuals from birth to age 50. It is initialised with the 2021 baseline population of 18 620 797.^20^ Although the entire 0–49 age range is modelled, our inferences are limited to the most relevant subgroups for each fracture: adults (18 years and older) for femoral shaft, patellar and humeral shaft fractures and children (18 years and younger) for PSCHF. Individuals exit the model on reaching age 50. The target subgroups align with the expert elicitation process, in which orthopaedic specialists provided parameter estimates for adults for the first three fracture types and for children for PSCHF. The model includes births and deaths as the only drivers of demographic change, excluding migration. Fertility rates, mortality rates and sex ratios at birth vary by age, sex and year and are consistently applied throughout the projection period. All parameters were adjusted according to the cycle length. Population updates and transitions between health states occur at the start of each cycle.

### Model assumptions

We assume that all incident cases of orthopaedic injury in the model represent persons presenting for treatment for the first time or who have been referred from a lower-level hospital. Additionally, all incident cases are assumed to involve a single new, closed, nondisplaced fracture; for patellar fractures, we further assume the extensor mechanism is intact. Cases requiring repeat surgery (in the ongoing disability state) are assumed to undergo the follow-up procedure only once. We also assume that risks, including incidence and treatment effectiveness, remain constant over time, with no ‘rebound’ effect, meaning that any health gains achieved are maintained. Furthermore, for modelling purposes, we assume that the probability of health-related events is independent of past events (the model does not account for fracture history); for example, a previous fracture does not increase the likelihood of sustaining another fracture. This reflects both the Markov memoryless assumption and the lack of setting-specific data on repeat fractures.

### Model data sources

We used data from multiple sources to develop age- and sex-specific projections of fracture prevalence and incidence in Malawi within population and Markov models. The analysis was based on 2021 base population data and sex- and age-specific mortality rates, fertility rates and sex ratios at birth from the UN Population Data Portal,^20^ as well as fracture prevalence and incidence estimates from the Global Burden of Disease (GBD) Database.^21^

We adjusted the fracture treatment costs from Twea *et al*^22^ by estimating total costs per case, which involved adding all procedure costs and multiplying the elicited average length of stay for each treatment type by the estimated daily admission costs. The procedure costs and daily admission costs for the interventions were calculated using the time-driven activity-based costing (TDABC) method, using both direct and indirect costs. More detailed information on the costing methodology can be found in the paper by Twea *et al*.^22^ We assigned a small nominal cost to the no-treatment arm, assuming that most expenses are indirect and borne by the patient, such as out-of-pocket spending on pain medication, even when they do not formally seek care. We used disability weights from the GBD study^21^ to quantify health effects. Table 1 summarises the model inputs.

**Table 1:** Model inputs with distribution and source of variation

Parameter types	Femur shaft	Patellar	Humeral Shaft	PSCHF	Distribution	Source of variation
No treatment costs						
Fracture	2.15 (1.075–3.225)	2.15 (1.075–3.225)	2.15 (1.075–3.225)	2.15 (1.075–3.225)	Gamma	Author assumption (±50%)
Ongoing disability	0	0	0	0	Gamma	Author assumption (±50%)
Surgery costs						
Fracture	478 (239–716)	197 (186–212) 231	231 (228–235)	173 (87–260)	Gamma	Author assumption (±50%)
Ongoing disability†	380 (343–434)	76 (38–113)	100 (50–149)	10 (5–15)	Gamma	Author assumption (±50%)
Conservative costs‡						
Fracture	1090* (1083–1099)	129 (121–134)	62 (57–69)	123 (116–128)	Gamma	Author assumption (±50%)
Ongoing disability	478 (432–546)	197 (186–212)	231 (228–235)	173 (170–177)	Gamma	Author assumption (±50%)
Disability weights*						
Fracture	0.11 (0.074–0.156)	0.05 (0.032–0.075)	0.035 (0.021–0.053)	0.035 (0.021–0.053)	Beta	GBD 2013 study (IHME)
Ongoing disability	0.11 (0.074–0.156)	0.05 (0.032–0.075)	0.035 (0.021–0.053)	0.035 (0.021–0.053)	Beta	GBD 2013 study (IHME)
Stable disability	0.042 (0.027–0.063)	0.055 (0.036–0.081)	0.035 (0.021–0.053)	0.035 (0.021–0.053)	Beta	Beta GBD 2013 study (IHME)
Chronic disability	0.042 (0.027–0.063)	0.055 (0.036–0.081)	0.035 (0.021–0.053)	0.035 (0.021–0.053)	Beta	GBD 2013 study (IHME)
No treatment transition probabilities						
Full function	0.041 (0.018–0.068)	0.14 (0.012–0.19)	0.171 (0.13–0.2)	0.15 (0.11–0.20)	Beta	Expert elicitation
Ongoing disability	0.774 (0.69-0.85)	0.81 (0.74–0.84)	0.70 (0.61–0.79)	0.69 (0.63–0.74)	Beta	Expert elicitation
Stable disability	0.167	0.039	0.12	0.14	Beta	Expert elicitation
Conservative transition probabilities*						
Full function	0.18 (0.12–0.24)	0.68 (0.60–0.75)	0.77 (0.70–0.85) 0	0.57 (0.50–0.65)	Beta	Expert elicitation
Ongoing disability	0.61 (0.50–0.70)	0.23 (0.18–0.28)	0.12 (0.08–0.16)	0.21 (0.16–0.26)	Beta	Expert elicitation
Stable disability	0.19 (0.14–0.24)	0.08 (0.05–0.11)	0.1 (0.07–0.13)	0.21 (0.16–0.26)	Beta	Expert elicitation
Surgery transition probabilities*						
Full function	0.87 (0.80–0.94)	0.81 (0.74–0.88)	0.89 (0.82–0.96)	0.87 (0.80–0.94)	Beta	Expert elicitation
Ongoing disability	0.05 (0.03–0.07)	0.06 (0.04–0.08)	0.03 (0.02–0.05)	0.04 (0.03–0.06)	Beta	Expert elicitation
Stable disability	0.07 (0.05–0.09)	0.12 (0.09–0.15)	0.06 (0.04-0.08)	0.08 (0.06–0.10)	Beta	Expert elicitation

* In the absence of treatment-specific disability weights, we assume state-specific weights are constant across treatment options; differences between treatments are propagated through their different transition probabilities.

† Due to limited data on cost variation by initial treatment option, we assume a constant cost for the second surgery; costs presented here are weighted based on the probability of second-line treatment, with lower values driven by low re-surgery rates.

‡ Conservative treatment incurs higher costs than surgical treatment due to a longer length of hospital stay.

As no studies used the same patient pathway, there were no transition probabilities available in the literature. The transition probabilities in the model were therefore obtained through a structured expert elicitation process with orthopaedic surgeons and clinical officers practising in tertiary hospitals. The protocol and results of the elicitation process are detailed in online supplemental appendix section S1. During the same elicitation, we also obtained estimates for treatment duration, transition probabilities, treatment effects and length of hospital stay. The transition probability inputs, the costs and the disability weights (which were not varied by pathway) are reported in online supplemental appendix table S1.

### Intervention options

We assessed the cost-effectiveness of three treatment strategies: no treatment, where no patients receive fracture care; conservative treatment only, where all patients receive conservative management; and surgical treatment only, where all patients receive surgical intervention. We first compare the cost-effectiveness of each treatment strategy to no treatment, then assess the relative cost-effectiveness of surgical treatment compared with conservative management.

### Model validation

Model validation was conducted through expert review and data cross-checking rather than formal external validation, as no published models of comparable scope were available for Malawi or similar settings. Input parameters derived from expert elicitation were compared with available empirical data where possible to assess plausibility, and the overall model structure, assumptions and outputs were reviewed for face validity by orthopaedic surgeons.

### Addressing uncertainty

To assess the impact of parameter uncertainty on model outcomes, we conducted both one-way and probabilistic sensitivity analyses (PSA) on the base-case scenarios to assess uncertainty in model outcomes. Online supplemental appendix table S1 summarises the input parameters, including their variation ranges, distributional assumptions and sources. In both sensitivity analyses, we varied key parameters including disability weights, transition probabilities, discount rates and incidence rates. For the PSA, we ran the model 1000 times and used the incremental net monetary benefit (INMB) framework to estimate the probability of cost-effectiveness under uncertainty. For each treatment comparison, we report the proportion of simulations with a positive INMB across a range of WTP thresholds.

### Scale-up scenarios

**Figure 2:**
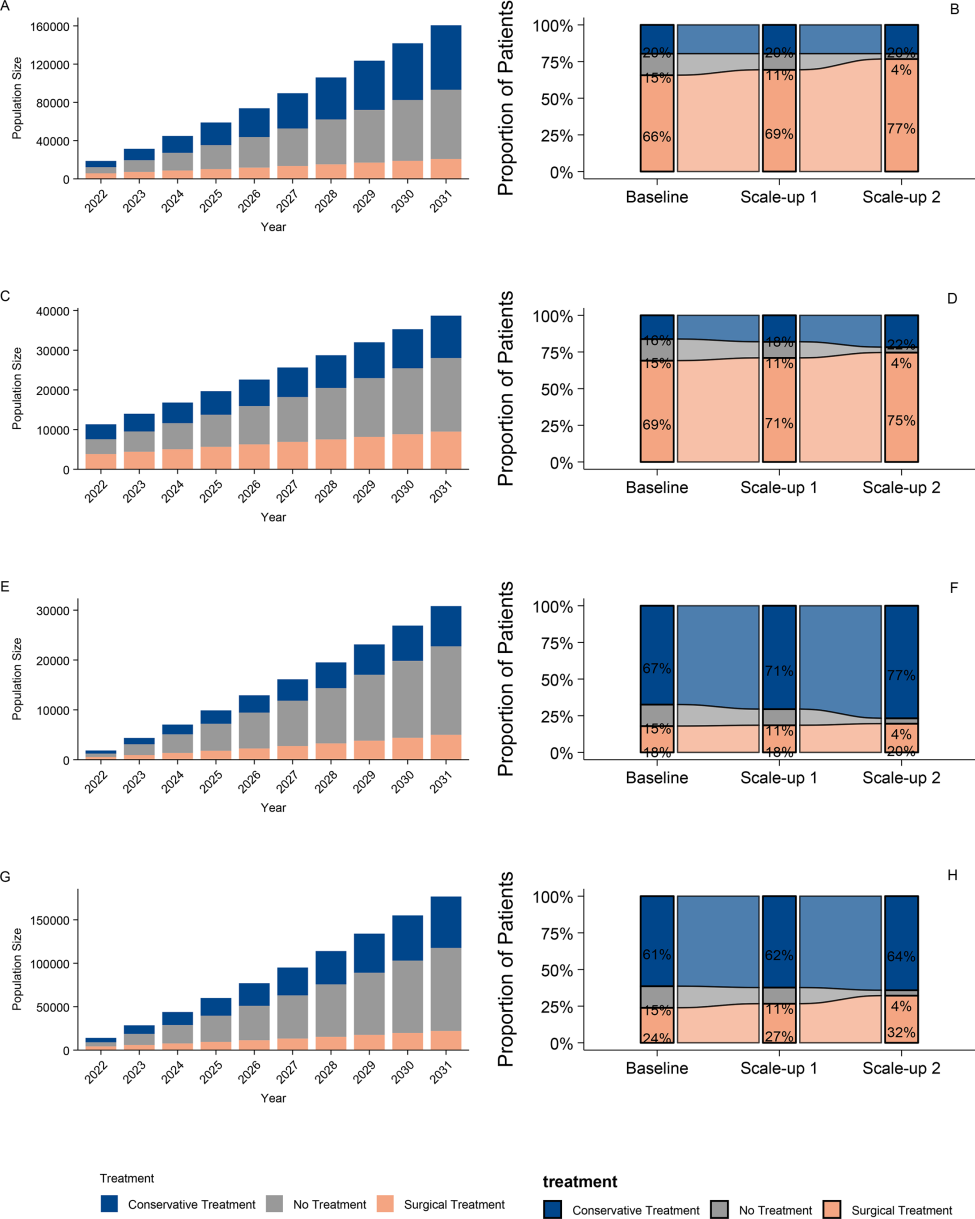
Projected burden of chronic stable disability and treatment distribution across scale-up scenarios for the four fracture types.

We include a status quo scenario that reflects current clinical practice, in which patients are distributed across the three treatment pathways based on elicited probabilities. The proportion of untreated patients is informed by estimates from Ngoie *et al*,^12^ while the distribution between conservative and surgical care is based on expert opinion. To explore the potential impact of expanding surgical capacity, we defined two scenarios for scale-up. In the first scenario, the proportion of patients receiving no treatment is reduced by 25%, with the difference reallocated between conservative and surgical treatment based on their estimated probability of being cost-effective. In the second scenario, the no-treatment group is reduced by 75%, with reallocation applied in the same manner. These adjustments are informed by the probabilities of cost-effectiveness estimated in the base-case PSA and illustrated in the figure 2 (plots B, D, F, H).

### Ethical considerations

The National Committee on Research in the Social Sciences and Humanities granted ethical approval for the study (Reference Number: P.07/22/657), while each hospital’s research committee granted site-specific approval.

We evaluated our study using the Consolidated Health Economic Evaluation Reporting (CHEERS) Checklist in online supplemental appendix section S2

### Patient and public involvement

Patients or the public were not involved in the design, conduct, reporting or dissemination plans of this research.

## Results

### Health impact and cost-effectiveness of no treatment relative to conservative management and surgical intervention

For conservative treatment, the highest average cost per person treated was $1200 for femur fractures, while the lowest was for humerus shaft fractures at $78. In comparison, surgical treatment was consistently more expensive than conservative treatment, except for femur fractures, where extended hospital stays made conservative care more costly. The highest surgical treatment cost was for femur fractures and the lowest for PSCHF. The cost of no treatment was comparable across fractures due to consistent cost assumptions applied in that scenario. In terms of health burden, the average DALYs per person in the absence of treatment were highest for patellar fractures (0.32) and lowest for humeral shaft fractures (0.11). The higher cumulative DALYs for patellar fractures compared with femoral shaft fractures reflect state occupancy trends (online supplemental appendix figure S1), with more patients with patellar fractures remaining in the chronic state, partly due to lower second-line treatment rates and fewer transitioning to full recovery over the simulation period. Differences in disability weights also contribute to femoral shaft fracture patients in the chronic state having lower weights, whereas patellar fracture patients experience a slight increase. Across all fractures, years of life lost (YLLs) were consistently much lower than years lived with disability (YLDs) and only for femur fractures were YLLs a significant share of DALYs.

Pairwise comparisons between the intervention approaches and no treatment also show variation in cost-effectiveness across fracture types (table 2). All treatment options were cost-increasing but health-improving compared with not providing treatment. The most significant health gains from surgical treatment relative to conservative care are observed for femur fractures and PSCHF. At the same time, smaller differences are seen for patellar and humeral shaft fractures. This pattern is reflected in the ICERs: surgical treatment yields more favourable cost-effectiveness for femur and PSCHF but results in a higher cost per DALY averted for patellar and humeral shaft fractures. Online supplemental appendix table S2 presents the cost-effectiveness of surgical interventions compared with conservative management across the fracture types. The results show that surgery is dominant for femoral shaft fractures (ICER: –US$6900 per DALY averted). For other fracture types, the ICERs are US$430 per DALY averted for PSCHF, US$1600 for patellar fractures and US$8,800 for humeral fractures.

**Table 2:** Cost-effectiveness analysis results per person by fracture type and treatment strategy

Fracture	Measure	Intervention approach		
		No treatment	Conservative treatment	Surgical treatment
Femur	Years lived with disability	0.23 (*0.14–0.34*)	0.21 (*0.13–0.31*)	0.1 (*0.061–0.15*)
	Years of life lost	0.0075 (*0.0075–0.0075*)	0.0039 (*0.0039–0.0039*)	0.0021 (*0.0021–0.0021*)
	Disability-adjusted life years	0.23 (*0.13–0.34*)	0.21 (*0.15–0.32*)	0.1 (*0.063–0.15*)
	Costs, thousand USD	0.0033 (*0.00098–0.0079*)	1.2 (*0.26–3.9*)	0.43 (*0.04–1.4*)
	Incremental DALYs averted	NA	0.02 (*0.013–0.028*)	0.13 (*0.078–0.02*)
	Incremental costs, thousand USD	NA	1.2 (*0.26–3.9*)	0.43 (*0.038–1.4*)
	ICER, thousand USD	NA	60 (*14.0–220*)	3.3 *(0.025-12*)
Patellar	Years lived with disability	0.32 (*0.2–0.62*)	0.22 (*0.12–0.38*)	0.20 (*0.11–0.35*)
	Years of life lost	0.00098 (*0.00098–0.00098*)	0.00069 (*0.00069–0.00069*)	0.0006 (*0.0006–0.0006*)
	Disability-adjusted life years	0.32 (*0.2–0.62*)	0.22 (*0.12–0.38*)	0.2 (*0.11–0.35*)
	Costs, thousand USD	0.0034 (*0.00071–0.0083*)	0.15 (*0.033–3.45*)	0.18 (*0.027–1.65*)
	Incremental DALYs averted	NA	0.1 (*0.0078–0.24*)	0.12 (*0.089–0.27*)
	Incremental costs, thousand USD	NA	0.15 (*0.029–0.44*)	0.18 (*0.025–0.64*)
	ICER, thousand USD	NA	1.4 (*0.21–4.1*)	1.4 (*0.18–3.8*)
Humeral Shaft	Years lived with disability	0.11 (*0.056–0.17*)	0.055 (*0.029–0.087*)	0.04 (*0.021–0.064*)
	Years of life lost	0.00093 (*0.00093–0.00094*)	0.00064 (*0.00064–0.00064*)	0.0011 (*0.0011–0.0011*)
	Disability-adjusted life years	0.11 (*0.057–0.17*)	0.056 (*0.029–0.088*)	0.041 (*0.022–0.065*)
	Costs, thousand USD	0.0032 (*0.00099–0.0075*)	0.078 (*0.019–3.23*)	0.21 (*0.022–1.82*)
	Incremental DALYs averted	NA	0.052 (*0.028–0.087*)	0.067 (*0.035–0.11*)
	Incremental costs, thousand USD	NA	0.074 (*0.016–0.23*)	0.2 (*0.019–0.81*)
	ICER, thousand USD	NA	1.4 (*0.32–5.0*)	3.0 (*0.26–11*)
PSCHF	Years lived with disability	0.13 (*0.078–0.23*)	0.085 (*0.052–0.15*)	0.039 (*0.023–0.069*)
	Years of life lost	0.0041 (*0.0041–0.0041*)	0.00055 (*0.00055–0.00055*)	0.00049 (*0.00049–0.00049*)
	Disability-adjusted life years	0.14 (*0.082–0.24*)	0.086 (*0.052–0.15*)	0.04 (*0.023–0.07*)
	Costs, thousand USD	0.0031 (*0.00018–0.006*)	0.14 (*0.052–0.15*)	0.16 (*0.023–0.07*)
	Incremental DALYs averted	NA	0.051 (*0.03–0.084*)	0.097 (*0.053–0.17*)
	Incremental costs, thousand USD	NA	0.13 (*0.028–0.47*)	0.15 (*0.019–0.49*)
	ICER, thousand USD	NA	2.6 (0.48–8.8)	1.6 (*0.18–6.8*)

All figures rounded to two significant digits. Uncertainty intervals, shown in italics, represent the minimum and maximum values derived from PSA results.

ICERS for both conservative and surgical treatments are calculated using no treatment as the reference. Online supplemental appendix table S2 number presents a comparison between conservative and surgical treatment.

CT, conservative treatment; ICER, incremental cost-effectiveness ratio; NT, no treatment; PSCHF, paediatric supracondylar humerus fractures; ST, surgical treatment.

### Sensitivity analysis

**Figure 3:**
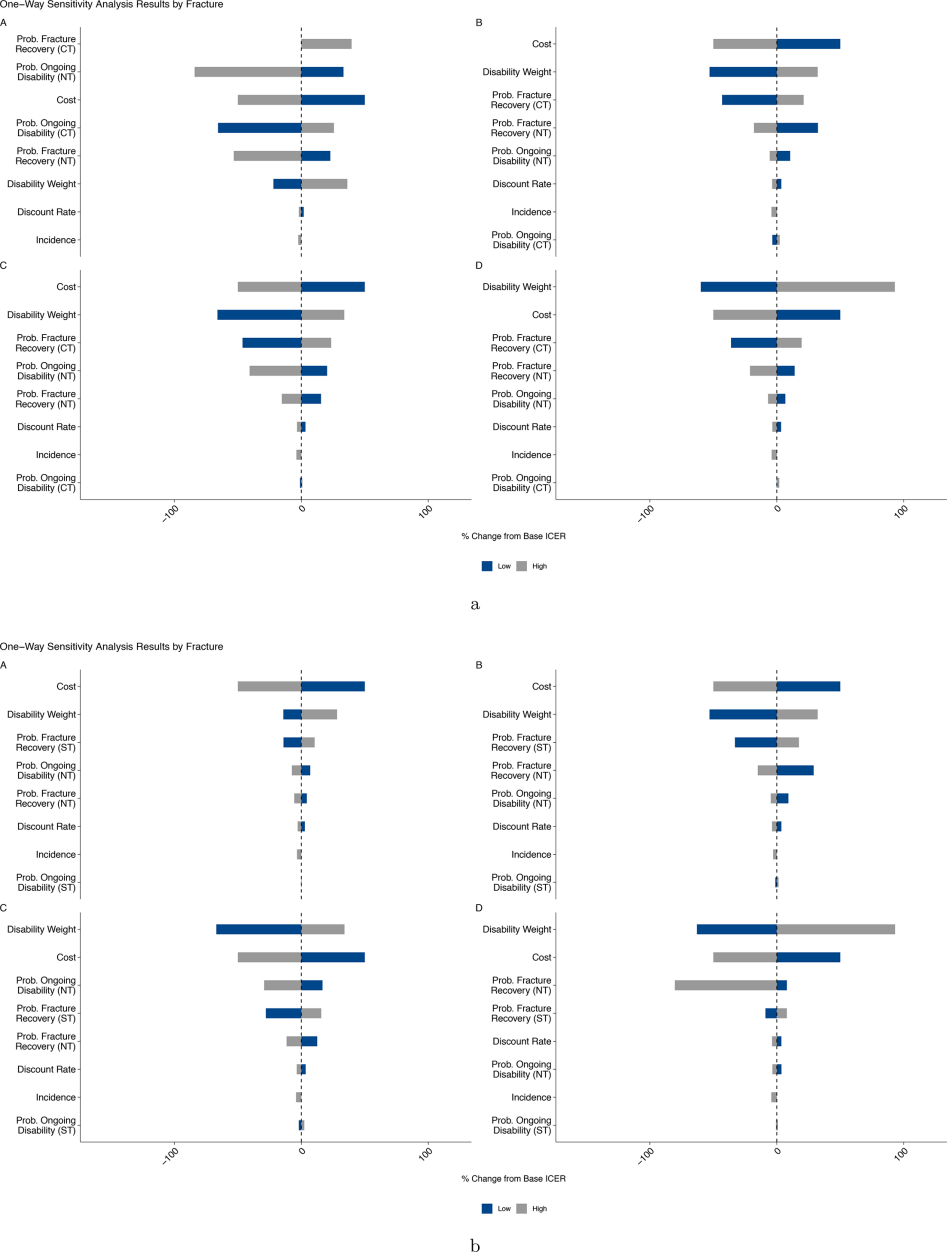
One-way sensitivity analysis results show the percentage change in the base-case ICER when comparing treatment strategies to no treatment across four fracture types. Panel (**A**) compares conservative treatment (CT) with no treatment (NT); panel (**B**) compares surgical treatment (ST) with no treatment (NT). Within each panel, sub-panels (a–d) correspond, respectively, to femoral shaft, patellar, humeral shaft and paediatric supracondylar humerus fractures. CT, conservative treatment; NT, no treatment; ST, surgical treatment. Parameter ranges and deterministic values used in the analysis are presented in online supplemental appendix table S1.

In the one-way sensitivity analysis, the most influential parameters on the ICERs varied by treatment strategy and fracture type (figure 3). For conservative treatment, cost inputs consistently had the greatest impact, contributing over 50% to ICER variation across all fractures. For surgical treatment, disability weights were the main driver of variation (30% or more), particularly for supracondylar humerus and patellar fractures, but were less influential for femur fractures. Variations in discount rates and incidence rates had comparatively minor effects, contributing less than 5% to ICER variation in both strategies. Figure 3 is a tornado plot that summarises the results of the one-way sensitivity analysis.

We used the PSA results to show the distribution of incremental costs and effects for each treatment option compared with no treatment, as illustrated in online supplemental appendix figure S2. The probability of each intervention being cost-effective for each pairwise comparison is reported in online supplemental appendix table S3. These results show that while treatment is not cost-effective at Malawi’s official WTP threshold, it becomes cost-effective at a threshold equivalent to one time the GDP per capita for some fractures. At a threshold of three times the GDP per capita, treatment is cost-effective across all fracture types, supporting clearer prioritisation decisions.

### Population impact and fracture-related costs with the intervention options

The model predicted varying fracture volumes and progression patterns across fracture states and types. As incidence rates were constant, the total incident population growth was primarily driven by population growth and comparable across fractures. Femur fractures had the highest increase in incident cases, while the other fracture types showed similar growth trends across the treatment options. The prevalent populations, those in the chronic disability state, also varied by fracture type, with humeral fractures showing the greatest increase and patellar fractures the lowest. There were differences across the treatment options, but the no treatment scenario consistently resulted in the highest prevalent population, followed by conservative and then surgical treatment, as shown in figure 2 (plots A, C, E, G).

Mortality patterns also varied across fractures. Notably, most of the disease burden from fractures was attributable to morbidity rather than mortality. While the model predicts some fracture-specific deaths, these were low across all fracture types. Online supplemental appendix figure S3 shows the total number of fracture deaths and YLLs by age for femoral shaft fractures.

### Health impact and costs of scaling up surgery relative to the status quo

We used the probability of cost-effectiveness for each treatment intervention as a scaling factor to increase access in the model. The specific scaling levels are shown in figure 2 (plots B, D, F, H). Under the current intervention coverage scenario, the model predicted approximately 610 DALYs averted under Scale-up Scenario 1 and approximately 1,800 under the more ambitious Scale-up Scenario 2. The more ambitious scale-up scenario resulted in an approximately 10% reduction in total DALYs relative to current intervention coverage, with total deaths decreasing from approximately 300 to 200 and YLLs from approximately 310 to 240. These health gains were associated with incremental costs of approximately US$1.7 million and US$5 million under Scale-up Scenarios 1 and 2, respectively, as shown in table 3.

**Table 3:** Cost-effectiveness analysis results

		Scenario		
	Measure	Current intervention coverage	Scale up (1)	Scale up (2)
All fractures	Years lived with disability	19 000	19 000	18 000
	Years of life lost	310	290	240
	Total deaths	300	270	200
	Disability adjusted life years	19 000	18 000	17 000
	Costs, million USD	44.0	46.0	49.0
	Difference in DALYs	NA	610	1800
	Difference in costs, million USD	NA	1.7	5.0
	Relative reduction in total disability adjusted life years (DALYs), %	NA	5.3	10.5

All figures rounded to two significant digits.

DALYs=years lived with disability+years of life lost.

## Discussion

This economic evaluation estimated the costs and health outcomes of treatment strategies for four orthopaedic fracture types: femoral shaft, humeral shaft, PSCHF and patellar, managed at the tertiary level within Malawi’s health system. To the best of our knowledge, this is the first study to model the population-level impact of orthopaedic treatment decisions, quantifying the health burden of untreated fractures in terms of DALYs. Our analysis of femur fracture cost-effectiveness allows for comparisons with prior studies in similar settings, while our estimates for the other three fracture types represent new contributions to the cost-effectiveness evidence base in low-income countries.

Regarding cost-effectiveness, variations exist across fracture types, driven by differences in incidence, baseline treatment distributions, relative treatment effectiveness and associated costs. For femoral shaft fractures, surgical intervention dominates due to the high incidence, significant differences in effectiveness between surgical and conservative care and lower surgical costs relative to conservative treatment. In contrast, the benefit of expanding surgical services is smaller for patellar fractures, with modest differences in treatment effect where the extensor mechanism is still intact, and surgical coverage is already high due to simpler surgical techniques and implant needs. A similar pattern is observed for the humeral shaft and PSCHF, where the cost-effectiveness of surgical expansion is also less pronounced. This is attributed to lower incidence, higher surgical costs and smaller differences in treatment effectiveness, particularly for humeral fractures. In our model, the impact of scaling up treatment is largely driven by incidence, with greater effects observed for fractures that are both more common and have larger differences in treatment effect size. While the overall impact is substantial under our assumed levels of no treatment (5–10%), the findings also underscore the importance of concurrent investments in injury prevention, particularly in road safety, alcohol control and other modifiable risk factors.^23^

We found that none of the interventions was cost-effective at Malawi’s official WTP threshold of $65 per DALY averted, which is very conservative and inevitably steers resource allocation towards less expensive, primary healthcare-level interventions. For fracture treatment, where unit costs are often several magnitudes higher than the threshold, even effective interventions like those included in this study tend to exceed it. At higher WTP thresholds, such as one to three times the GDP per capita, the probability of orthopaedic care being cost-effective increases. Although the macroeconomic and fiscal situation in Malawi is very challenging, the country still needs to build capacity for the appropriate management of common health concerns, including road injuries, in the most cost-effective manner possible. This may justify targeted investments in areas such as essential injury or cancer care that are not currently cost-effective but are expected to become so as national income rises. Those nuanced discussions extend far beyond the scope of this study, but this study provides evidence to help inform and frame those discussions.

Our findings build on a limited body of economic evaluations of orthopaedic fracture care in low-income settings. Prior studies have primarily focused on femoral shaft fractures^7 24–26^ and have typically been cost-utility analyses using QALYs, rather than cost-effectiveness analyses based on DALYs, as in our study. In addition, these earlier models were static rather than dynamic, further limiting direct comparability.^7 24–26^ Other research has largely been limited to cohort studies that assess functional outcomes and patient-reported measures.^27^ For humeral shaft, patellar and PSCHF fractures, we found no published cost-effectiveness analyses in resource-constrained settings. However, studies reporting costs, epidemiology and clinical outcomes, including complication rates, return to function and radiological results, were available and used to validate our model parameters.^9 28–31^

Our study has several limitations that may affect the accuracy and generalisability of our findings. First, our estimates of the proportion of patients treated at tertiary hospitals are based on an epidemiological study that did not include all eligible hospitals^14^ and may be overestimated. We assumed this proportion was constant across fracture types, which may not accurately reflect variations in treatment patterns. Where inputs were not disaggregated by fracture type, we used trends from the same study to estimate the distribution of specific fractures. These assumptions may limit the precision of our incidence and prevalence estimates. Second, because the cause of fractures in adults (eg, whether they were low-impact, osteoporosis-related fractures or high-impact injuries) was not consistently recorded in patient files, we did not distinguish between these types, which may affect how well the results apply to different fracture causes. Third, one elicited parameter, outcomes for untreated patients, was based on variables not directly observed by clinicians, potentially introducing errors. Follow-up treatment rates were also based on clinician recall and may be right-skewed, as patients often seek follow-up at local facilities rather than returning to tertiary hospitals. This lack of continuity and follow-up tracking may bias these estimates. Additionally, we assumed that the cost of follow-up surgeries equalled that of initial surgeries, which may not be true in all cases. Finally, complication rates were informed by clinician-weighted estimates across fracture types, though our model did not fully capture variation by fracture. To mitigate these issues, we communicated underlying assumptions during elicitation to ensure estimates were contextually grounded.

## Conclusion

Our study supports previous research indicating that surgery is generally more cost-effective than conservative treatment for orthopaedic fractures, particularly femur fractures. However, we significantly contribute to previous studies by systematically examining four common types of orthopaedic injuries and their corresponding surgical and non-surgical responses, enabling a more comprehensive assessment of value for money in orthopaedic care. The findings highlight the importance of considering local epidemiological and health system factors when prioritising orthopaedic surgery services. They also lay the groundwork for understanding the implications of scaling up orthopaedic fracture care and point to the potential value of preventive interventions and the need for further economic evaluations of other orthopaedic injuries.

## Supplementary material

10.1136/bmjgh-2025-022014 online supplemental file 1

10.1136/bmjgh-2025-022014 online supplemental figure 1

10.1136/bmjgh-2025-022014 online supplemental figure 2

10.1136/bmjgh-2025-022014 online supplemental figure 3

10.1136/bmjgh-2025-022014 online supplemental file 2

## Data Availability

Data are available in a public, open access repository. Data are available upon reasonable request.
