# Supplementary material for: Cost-effectiveness of surgical and conservative interventions for orthopaedic injuries in Malawi: a modelling study
Source: BMJ Glob Health. 2026 Aug 14;11(8):e022014. doi: 10.1136/bmjgh-2025-022014 (PMC13479495; doi:10.1136/bmjgh-2025-022014)
Supplement: Supplementary file 1 [file bmjgh-11-8-s001.pdf]

BMJ Global Health Author Reflexivity Statement

Adapted from Morton, B., Vercueil, A., Masekela, R., Heinz, E., Reimer, L., Saleh, S., Kalinga, C., Seekles, M., Biccard, B., Chakaya, J., Abimbola, S., Obasi, A. and Oriyo, N. (2022), Consensus statement on measures to promote equitable authorship in the publication of research from international partnerships. Anaesthesia, 77: 264-276. https://doi.org/10.1111/anae.15597

**Study conceptualisation**	
1. How does this study address local research and policy priorities?	This research was based on an understanding of the gaps in cost-effectiveness information for the review of health benefit packages. It was noted that there is very little evidence for surgical conditions, and given the increasing incidence of orthopaedic conditions, we chose to focus on these to help fill the knowledge gap and contribute to the evidence base considered when reviewing health benefit packages. The study focused on tertiary hospitals to address the lack of cost and cost-effectiveness evidence at that level.
2. How were local researchers involved in study design?	The first author is local and was primarily involved in designing the study to ensure that it addresses key evidence gaps.
**Research management**	
3. How has funding been used to support the local research team(s)?	The first author is a PhD candidate and was funded through this study, which supported their time on the research as well as data collection activities and the involvement of a local research assistant.
Data acquisition and analysis	
4. How are research staff who conducted data collection acknowledged?	The first author conducted the data collection, and a local research assistant was also supported through the same program.
5. How have members of the research partnership been provided with access to study data?	The study data was made available to the author team for the purposes of reviewing and validating the research methods and analysis, in line with data protection and confidentiality requirements.
6. How were data used to develop analytical skills within the partnership?	As this study was conducted as part of a PhD, the first author was responsible for the analysis, with guidance from the other authors. This process contributed to the development of critical analytical skills.
Data interpretation	
7. How have research partners collaborated in interpreting study data?	The full author team was involved in reviewing and interpreting the study findings.
Drafting and revising for intellectual content	
8. How were research partners supported to develop writing skills?	The first author was supported through formal training and led the development of the first draft of the manuscript.
9. How will research products be shared to address local needs?	The research outputs are planned to be shared with the participating hospitals and the Ministry of Health, including through reports and presentations to support decision-making.
Authorship	

10. How is the leadership, contribution and ownership of this work by LMIC researchers recognised within the authorship?	The first author is an LMIC researcher and led the execution of the analysis and the writing of the manuscript.
11. How have early career researchers across the partnership been included within the authorship team?	As the research design and execution were primarily led by the first author, opportunities for including additional early career researchers were limited.
12. How has gender balance been addressed within the authorship?	The first author is female, although overall gender balance within the authorship is limited.
Training	
13. How has the project contributed to training of LMIC researchers?	The manuscript forms part of the first author's PhD training.
Infrastructure	
14. How has the project contributed to improvements in local infrastructure?	The project did not directly contribute to improvements in local infrastructure, as its primary focus was on generating evidence.
Governance	
15. What safeguarding procedures were used to protect local study participants and researchers?	Ethical approval was obtained from the relevant institutional review boards. All data were handled in accordance with established confidentiality and data protection protocols, and no identifying information was included in the analysis.
