# Supplementary material for: Cost-effectiveness of surgical and conservative interventions for orthopaedic injuries in Malawi: a modelling study
Source: BMJ Glob Health. 2026 Aug 14;11(8):e022014. doi: 10.1136/bmjgh-2025-022014 (PMC13479495; doi:10.1136/bmjgh-2025-022014)
Supplement: Supplementary file 2 [file bmjgh-11-8-s002.pdf]

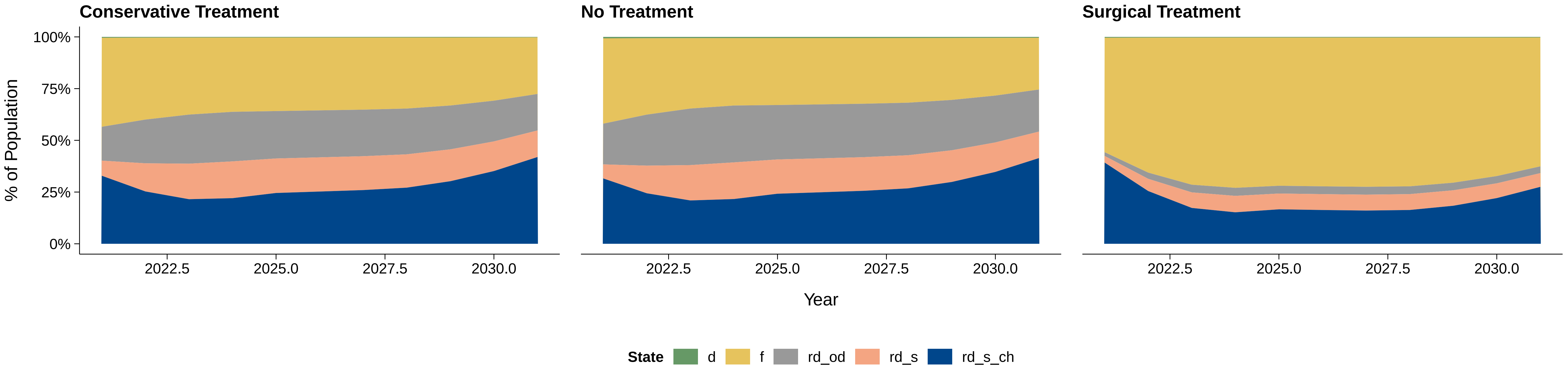

Conservative Treatment
No Treatment
Surgical Treatment
100%
75%
50%
25%
0%
% of Population
2022.5
2025.0
2027.5
2030.0
Year
State
d
f
rd_od
rd_s
rd_s_ch

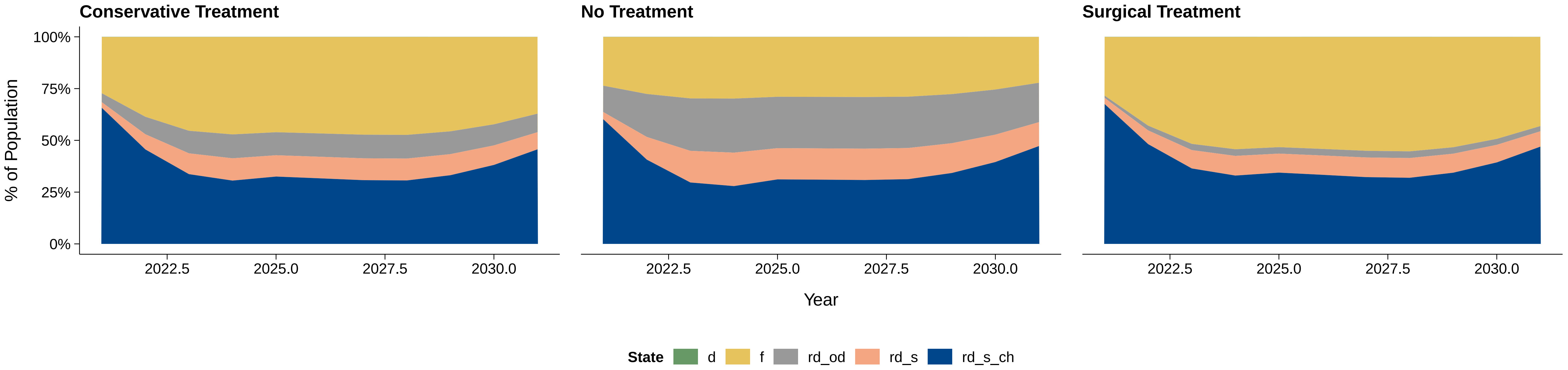

Conservative Treatment
No Treatment
Surgical Treatment
100%
75%
50%
25%
0%
% of Population
2022.5
2025.0
2027.5
2030.0
Year
State
d
f
rd_od
rd_s
rd_s_ch
