# Supplementary material for: Cost-effectiveness of surgical and conservative interventions for orthopaedic injuries in Malawi: a modelling study
Source: BMJ Glob Health. 2026 Aug 14;11(8):e022014. doi: 10.1136/bmjgh-2025-022014 (PMC13479495; doi:10.1136/bmjgh-2025-022014)
Supplement: Supplementary file 3 [file bmjgh-11-8-s003.pdf]

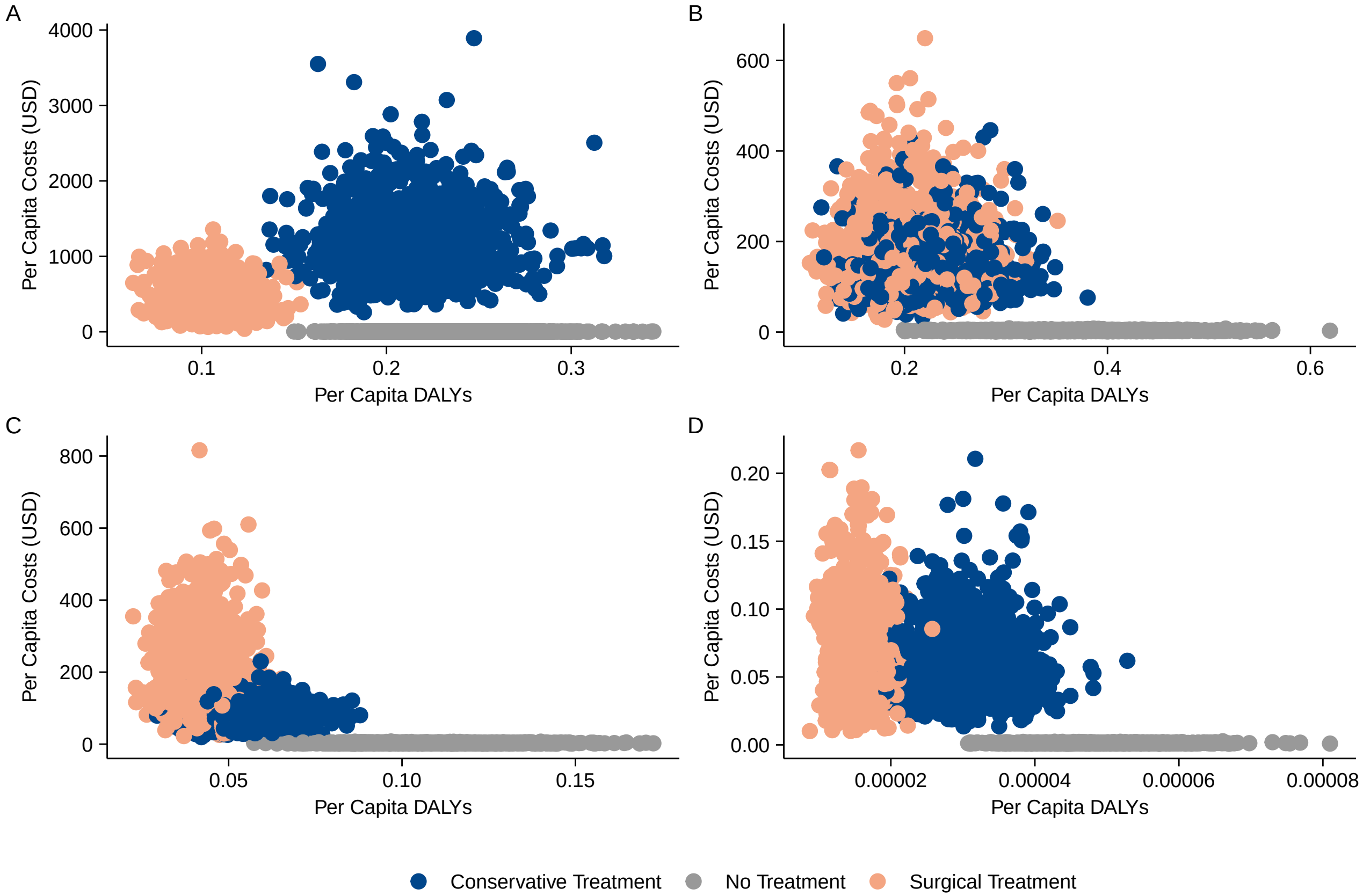

A
4000
3000
2000
1000
0
0.1
0.2
0.3
Per Capita Costs (USD)
Per Capita DALYs
B
600
400
200
0
0.2
0.4
0.6
Per Capita Costs (USD)
Per Capita DALYs
C
800
600
400
200
0
0.05
0.10
0.15
Per Capita Costs (USD)
Per Capita DALYs
D
0.20
0.15
0.10
0.05
0.00
0.00002
0.00004
0.00006
0.00008
Per Capita Costs (USD)
Per Capita DALYs
Conservative Treatment
No Treatment
Surgical Treatment
