# Supplementary material for: Cost-effectiveness of surgical and conservative interventions for orthopaedic injuries in Malawi: a modelling study
Source: BMJ Glob Health. 2026 Aug 14;11(8):e022014. doi: 10.1136/bmjgh-2025-022014 (PMC13479495; doi:10.1136/bmjgh-2025-022014)
Supplement: Supplementary file 4 [file bmjgh-11-8-s004.pdf]

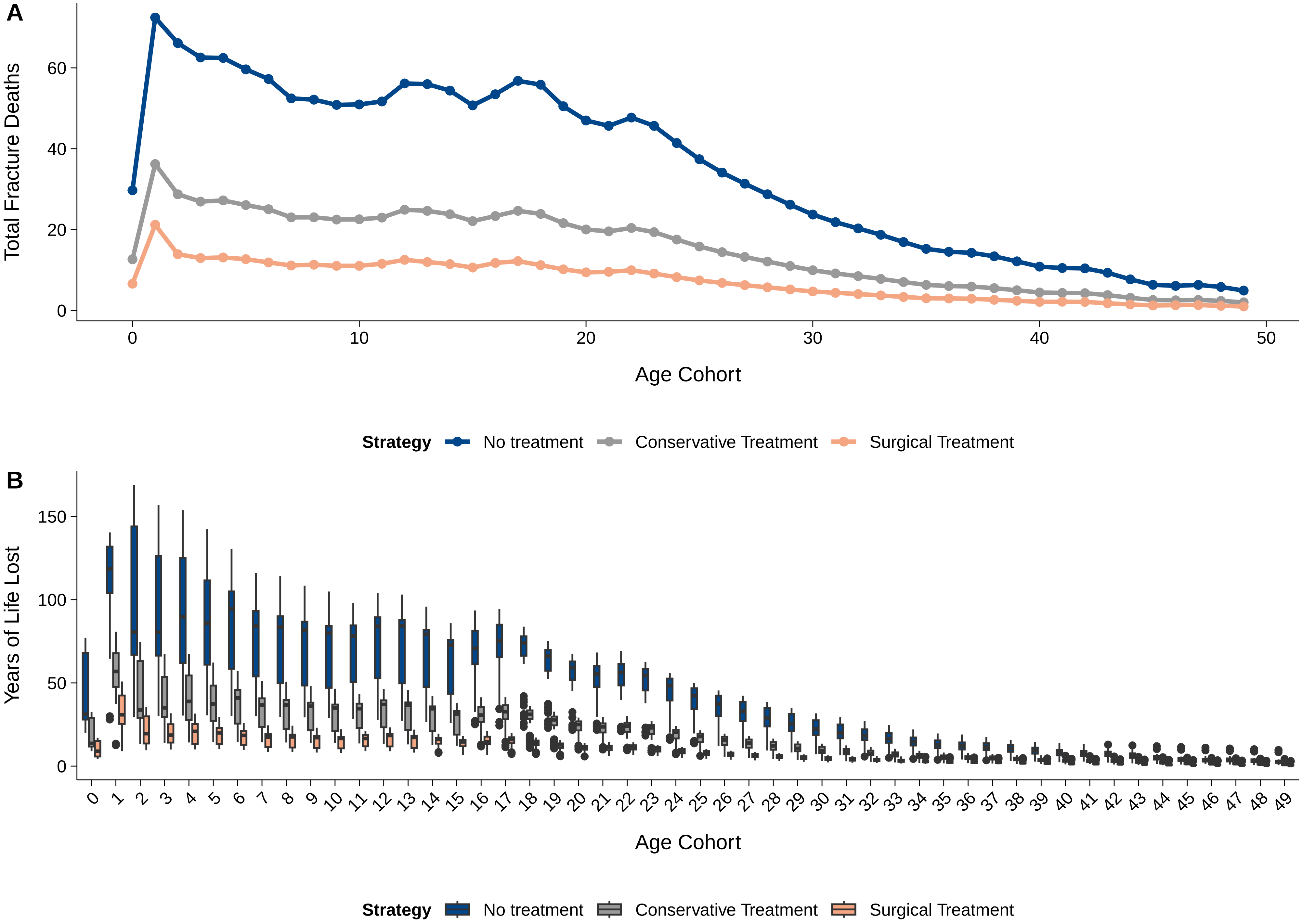
A
Total Fracture Deaths
0
20
40
60
0
10
20
30
40
50
Age Cohort
Strategy
No treatment
Conservative Treatment
Surgical Treatment
B
Years of Life Lost
0
50
100
150
0 1 2 3 4 5 6 7 8 9 10 11 12 13 14 15 16 17 18 19 20 21 22 23 24 25 26 27 28 29 30 31 32 33 34 35 36 37 38 39 40 41 42 43 44 45 46 47 48 49
Age Cohort
Strategy
No treatment
Conservative Treatment
Surgical Treatment
