# Supplementary material for: Cost-effectiveness of surgical and conservative interventions for orthopaedic injuries in Malawi: a modelling study
Source: BMJ Glob Health. 2026 Aug 14;11(8):e022014. doi: 10.1136/bmjgh-2025-022014 (PMC13479495; doi:10.1136/bmjgh-2025-022014)
Supplement: Supplementary file 5 [file bmjgh-11-8-s005.pdf]

Supplementary Materials

Section S1: Structured Expert Elicitation Protocol and Results

Background: The elicitation process aimed to elicit inputs into the health economic evaluation model. The choice of elicited inputs will be based on observed gaps in the literature for the diagnoses being evaluated. We will seek expert opinions on the progression rates between allowable transition states, time-to-event, and treatment effects. Progression rates have been categorised into three types: progressions with no treatment, conservative treatment, and surgical treatment.

Identification and Recruitment of Experts: Experts for the elicitation process will be selected primarily from the roster of specialist orthopaedic surgeons and clinical officers affiliated with five hospitals: Queen Elizabeth Central Hospital, Kamuzu Central Hospital, and Zomba Central Hospital. Given the limited number of experts in this field, we plan to initially contact all orthopaedic surgeons and clinical officers in the two hospitals, possibly extending to other hospitals if necessary. The author will initiate contact with these experts through phone calls or in-person visits to extend invitations for their participation in the survey. Bjoke et al. (1) suggested a range of four to twenty experts for structured expert elicitation studies. A key consideration was the heterogeneity of the experts in terms of their level of specialisation, geographical location, and clinical case mix experience (2), all of which would influence their judgement on the elicited estimates.

Elicitation Process: Upon accepting the invitation, experts will be engaged to coordinate a suitable time and date for the survey. The facilitator will introduce the survey by providing an overview of its purpose, application of its findings, objectives of the elicitation exercise, and a framework for extracting information. Given the low number of experts, the elicitation script will be piloted with only one expert. Each survey session is estimated to last approximately 1.5 to 2 hours per participant. The survey aims to gather expert opinions on the progression rates for each orthopaedic diagnosis, considering three possible alternatives. Experts will be given background information that includes the purpose, the use case of the elicited information, and the methodology of the elicitation process. The facilitator will guide the experts through the survey, prompting them to provide responses that will serve as a basis for the subsequent development of the Markov model. By adhering to this structured elicitation process, we aim to gather insights from experienced orthopaedic surgeons, enhancing the accuracy and reliability of the Markov model as we assess the cost-effectiveness of treatments for the specified orthopaedic diagnoses.

Structure of the questions: The questions about progression rates aim to gather expert opinions based on observed frequencies in clinical practice. They address progression rates for each treatment scenario, initial probabilities, and possible complications that affect the cost and effectiveness of treatment.

Methodology: The questions are consistent and involve a hypothetical group of 100 patients diagnosed with one of the orthopaedic conditions under study. Variable interval methods are used to improve the accuracy of expert input.

Elicitation Process:

For each parameter, experts will be asked to provide estimates as follows:

- Best Guess: The expert's most informed and educated estimate.
- Lower Limit: The minimum value they believe is plausible for the parameter.
- Upper Limit: The maximum value they consider plausible for the parameter.

This structured approach is designed to capture a range of perspectives, considering both the central tendency and potential variability in expert opinions. We address the uncertainty in clinical situations by keeping estimates within realistic ranges. Using variable interval methods and plausible quantiles helps us better understand expert opinions and improves our model's ability to estimate the cost-effectiveness of orthopaedic diagnoses accurately.

Combining the individual estimates: The elicited distributions from experts will be combined using the linear pooling method(3). We will assign equal weights to each expert and carry out the pooling process in Microsoft Excel. We will adjust for outlier responses based on a comparison with the literature. Inputs from the literature were extracted through a rapid review. All inputs will be calibrated based on literature values and insights from orthopaedic experts in similar settings.

Expert Response rate and participant characteristics: Eighteen orthopaedic specialists and orthopaedic clinical officers were contacted by phone or face-to-face. All 18 agreed to participate; of these, interviews were conducted with 11, as the others were either unavailable for the interview or later declined to participate.

References

1. Bojke L, Soares M, Claxton K, Colson A, Fox A, Jackson C, et al. Developing a reference protocol for structured expert elicitation in health-care decision-making: a mixed-methods study. Health Technol Assess. 2021 Jun;25(37):1–124. doi:10.3310/hta25370

2. Soares MO, Bojke L, Dumville J, Iglesias C, Cullum N, Claxton K. Methods to elicit experts' beliefs over uncertain quantities: application to a cost effectiveness transition model of negative pressure wound therapy for severe pressure ulceration. Stat Med. 2011 Aug 30;30(19):2363–80. doi:10.1002/sim.4288

3. Cooke R. Experts in uncertainty: opinion and subjective probability in science. Oxford university press; 1991.

Table S1: Model Inputs

Table S1: Model Parameter Inputs

Parameter Group	Parameter	Femur Fracture	Patella Fracture	Humerus Fracture	PSCHF	Data Sources
		Mean (CI)	Mean (CI)	Mean (CI)	Mean (CI)	
Probabilities	F →FFa	4.1 *(1.8 - 6.8)*	14.4 *(12 - 18.6)*	17.1 *(13.1 - 20)*	15.3 *(10.8 - 20.1)*	Expert Elicitation
	F →ODa	77.4 *(68.8 - 85.3)*	80.7 (73.6 - 83.9)	69.8 *(61.3 - 79.4)*	68.8 *(62.5 - 74.4)*	Expert Elicitation
	F →CSDa	0.8 *(0.05 - 2.1)*	0 *(0 - 0)*	0 *(0 - 0)*	0.05 *(0 - 0.15)*	Expert Elicitation
	F →FFb	17.9 *(13.1 - 23)*	68.7 *(60.4 - 76.1)*	77.2 *(70.3 - 83.9)*	57 *(49.7 - 63.7)*	Expert Elicitation
	F →ODb	61.4 *(54.4 - 67.8)*	22.7 (16.3 - 27.8)	11.9 *(8.8 - 15)*	20.7 *(17.7 - 22.9)*	Expert Elicitation
	F →CSDb	0.04 *(0.01 - 0.08)*	0 (0 - 0)	0 *(0 - 0)*	0 (0 - 0)	Expert Elicitation
	F →FFc	87.3 *(83 - 91.8)*	81.3 *(73.3 - 88.1)*	89.1 *(83 - 94.3)*	87.3 *(83 - 91.8)*	Expert Elicitation
	F →ODc	5 (3.2 - 7.9)	5.5 *(2.9 - 8.8)*	3.5 *(2.1 - 5.3)*	3.8 *(2.4 - 5.6)*	Expert Elicitation
	F →CSDc	0.3 *(0 - 0.8)*	0 *(0 - 0)*	0 *(0 – 0)*	0 *(0 - 0)*	Expert Elicitation
	Pr - Surgery	76.6 (67.5 - 82.8)	80.6 *(72.3 - 85.4)*	21.4 *(17.3 - 26.7)*	28.3 *(22.5 - 34.6)*	Expert Elicitation
	Pr - Re-operation	79.6 *(71 - 86.6)*	38.4 *(30.8 - 36.6)*	43.1 *(37 - 50.3)*	5.65 *(3.5 - 7)*	Expert Elicitation
Time Parameters	Surgery waiting time	18 *(8 - 30)*	9 *(5 - 15)*	25 *(18 – 33)*	6 *(3 - 9)*	Expert Elicitation
	Length of stayb	57 *(44 - 73)*	4 *(3 - 5)*	0 *(0 - 0)*	6 *(3 - 8)*	Expert Elicitation
	Length of stayc	18 *(9 - 33)*	7 *(4 – 11)*	7 (4 - 12)	5 *(3 - 7)*	Expert Elicitation
Epi. Parameters	Incidence (under 5)	58.4 *(45.4 - 75.5)*	159.4 *(131.6 - 201.1)*	85.1 *(66.6 - 109.5)*	85.1 *(66.6 - 109.5)*	GBD Study 2021 (18)
	Incidence (5-9)	48.6 *(36.7 - 48.6)*	213.4 *(158.9 - 285.3)*	101.9 *(74.3 - 138.1)*	101.9 (74.3 - 138.1)	GBD Study 2021 (18)
	Incidence (10 - 14)	54.7 *(42 - 71.2)*	246 *(178.6 - 337.5)*	116 *(84.2 - 154.1)*	116 (84.2 - 154.1)	GBD Study 2021 (18)
	Incidence (15 - 19)	64 *(51.1 - 81.9)*	237.3 *(178.8 - 313.9)*	114.4 *(85.9 - 151.9)*	114.4 *(85.9 - 151.9)*	GBD Study 2021 (18)
	Incidence (20 - 24)	65.6 *(51.6 - 84.7)*	217 *(169.8 - 280.7)*	105 *(81.2 - 135.3)*	105 *(81.2 - 135.3)*	GBD Study 2021 (18)
	Incidence (25 - 29)	56 (44 - 73.5)	175.5 *(134.4 - 228)*	85.2 *(63.9 - 116.8)*	85.2 *(63.9 - 116.8)*	GBD Study 2021 (18)
	Incidence (30 - 34)	50.3 *(38.7 - 66.8)*	150.9 *(115.6 - 199.4)*	73.2 *(55 - 100.5)*	73.2 *(55 - 100.5)*	GBD Study 2021 (18)
	Incidence (35 - 39)	51.2 *(38.8 - 70.5)*	145.6 *(111.8 - 190.2)*	70.8 *(52.7 - 95.2)*	70.8 *(52.7 - 95.2)*	GBD Study 2021 (18)
	Incidence (40 - 44)	54 *(41.4 - 71)*	143.2 *(115.7 - 179)*	69.7 *(63.1 - 89.9)*	69.7 *(63.1 - 89.9)*	GBD Study 2021 (18)
	Incidence (45 - 49)	62.9 *(48.3 - 83.2)*	156.3 *(193.7 - 123.9)*	76 *(58.4 - 98.7)*	76 *(58.4 - 98.7)*	GBD Study 2021 (18)
	Prevalence (under 5)	12.7 *(8.6 - 17.6)*	95.4 *(82.6 - 111.1)*	12.5 *(9 - 17.9)*	12.5 *(9 - 17.9)*	GBD Study 2021 (18)
	Prevalence (5-9)	16.1 *(12.5 - 20.1)*	239.1 *(201.7 - 284.4)*	15.7 *(10.8 - 22.1)*	15.7 *(10.8 - 22.1)*	GBD Study 2021 (18)
	Prevalence (10 - 14)	23.1 *(18.9 - 27.5)*	442.6 *(372.3 - 539.7)*	18.8 *(13.1 - 26.5)*	18.8 *(13.1 - 26.5)*	GBD Study 2021 (18)
	Prevalence (15 - 19)	32.7 *(27.9 - 38.3)*	674.8 *(579.4 - 789.3)*	20.1 *(15.2 - 27.4)*	20.1 *(15.2 - 27.4)*	GBD Study 2021 (18)
	Prevalence (20 - 24)	45.2 *(39.6 - 51.2)*	899.6 *(784.5 - 1031.1)*	20.5 *(15.7 - 26.4)*	20.5 *(15.7 - 26.4)*	GBD Study 2021 (18)
	Prevalence (25 - 29)	56.8 *(50.9 - 63.2)*	1133.4 *(1003.9 - 1285.4)*	19.6 *(15.5 - 25)*	19.6 *(15.5 - 25)*	GBD Study 2021 (18)
	Prevalence (30 - 34)	70.2 *(63.2 - 78)*	1347.3 *(1200.3 - 1518.3)*	19.6 *(15.5 - 25)*	19.6 *(15.5 - 25)*	GBD Study 2021 (18)
	Prevalence (35 - 39)	86.2 *(79.4 - 95)*	1572.7 *(1403.1 - 1750.7)*	21.6 *(17 - 26.2)*	21.6 *(17 - 26.2)*	GBD Study 2021 (18)
	Prevalence (40 - 44)	106.9 *(99.2 - 116.6)*	1841.6 *(1665.8 - 2029.8)*	24.1 (20.8 - 28.1)	24.1 *(20.8 - 28.1)*	GBD Study 2021 (18)
	Prevalence (45 - 49)	134.4 *(124.6 - 144.4)*	2141.7 *(1944.8 - 2346.4)*	28.4 *(24.5 - 32.8)*	28.4 *(24.5 - 32.8)*	GBD Study 2021 (18)

Table B.4: Model Parameter Inputs.
Note: Rates presented as incidence per 100,000 population
Incidence and Prevalence rates are presented as unadjusted as provided from the IHME GBD Database
Surgery waiting time is in days
Superscripts indicate the corresponding treatment strategies: a No treatment, b Conservative treatment, c Surgical treatment.

Figure S1: State Occupancy Plot

Figure S1: State occupancy over time for femur fractures (top) and patella fractures (bottom) during the simulation period.

Figure S2: Probability Sensitivity Analysis Plot

Figure S2: Cost Effectiveness Scatter Plot

This figure illustrates the findings of a probabilistic sensitivity analysis (PSA) that compares the incremental costs and DALYs averted for three treatment options: no treatment, conservative treatment, and surgical treatment. The analysis covers four types of fractures: (A) Femur fracture, (B) Patella fracture, (C) Humerus shaft fracture, and (D) PSCHF.

Figure S3: Mortality Trends Plot

Figure S3: Mortality Trends Plot.

Total years of life lost (Panel A) and total number of deaths (Panel B) due to femur fractures, by age group and treatment strategy in the base case scenarios.

Table S2: Cost-Effectiveness Results: Conservative vs Surgical Treatment

Incremental cost-effectiveness ratios (ICERs) comparing surgical to conservative treatment across fracture types.

**Fracture Type**	**ICER (US$ per DALY averted)**
Femoral Shaft	-6,900
Patella	1,600
Humerus	8,800
PSCHF	430

Table S1: Incremental cost-effectiveness ratios (ICERs) comparing surgical to conservative treatment across fracture types.

Table S3: PSA Summary Results

Fracture	WTP Threshold	Conservative vs No Treatment	Surgery Vs No Treatment	Surgery vs Conservative Treatment
Femoral Shaft	$65 per DALY	0 · 0	0 · 0	96 · 3
Patella		0 · 0	0 · 0	39 · 8
Humerus Shaft		0 · 0	0 · 0	8 · 5
PSCHF		0 · 0	0 · 0	46 · 6
Femoral Shaft	1*GDP per DALY	0 · 0	0 · 4	97 · 2
Patella		16 · 2	16 · 2	44 · 3
Humerus Shaft		4 · 6	1 · 3	10 · 8
PSCHF		0 · 1	4 · 2	56 · 3
Femoral Shaft	3*GDP per DALY	0 · 0	17 · 3	98 · 7
Patella		91 · 6	86 · 8	52 · 8
Humerus Shaft		75 · 5	22 · 3	15 · 7
PSCHF		13 · 0	50 · 6	73 · 1

Table S3: Proportion of PSA iterations in which each treatment strategy is cost-effective compared to a comparator, based on incremental net monetary benefit. Results are shown for three WTP thresholds: $65 per DALY averted, 1× GDP per capita, and 3× GDP per capita.
